# Supplementary material for: Microglial phagocytosis mediates long-term restructuring of spinal GABAergic circuits following early life injury
Source: Brain Behav Immun. 2023 Jul;111:127–37. doi: 10.1016/j.bbi.2023.04.001 (PMC11932970; doi:10.1016/j.bbi.2023.04.001)
Supplement: Supplementary data 3 [file mmc3.pdf]

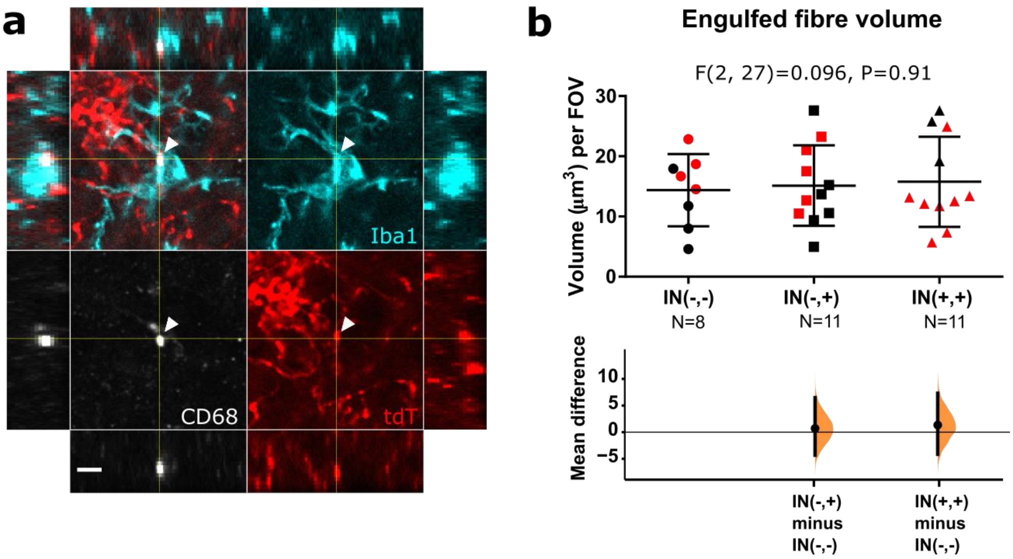

Figure S1. Adult incision did not alter A-fibre engulfment. a. Representative confocal image showing microglia (Iba1, cyan), lysosomes (CD68, grey), and A-fibres (tdT, red). White arrowheads point out overlap of Iba1, CD68, and tdT, indicating engulfment. Cross-hairs show position of the xz and yz side-view panels. Scale bar = 5μm. **b.** Engulfment of A-fibres is not altered following adult incision regardless of neonatal incision. Mean difference between adult incision only and naive animals (IN(-,+) minus IN(-,-)): 0.75 [95.00% CI -4.26, 6.52], mean difference between adult incision with neonatal incision and naive animals IN(+,+) minus IN(-,-)): 1.39 [95.00% CI -4.09, 7.36]. FOV = 192.74 x 192.74 x 50 μm. N-numbers and P-values are indicated in Figures. Black and red data points indicate females and males.
